# Oncologic and Operative Outcomes of Robotic Staging Surgery Using Low Pelvic Port Placement in High-Risk Endometrial Cancer

**DOI:** 10.3390/curroncol31120576

**Published:** 2024-12-05

**Authors:** Jeeyeon Kim, Jiheum Paek

**Affiliations:** Division of Gynecologic Oncology, Department of Obstetrics and Gynecology, Ajou University School of Medicine, Suwon 16499, Republic of Korea; jeeyeonkim211@gmail.com

**Keywords:** endometrial cancer, robotic surgery, lymphadenectomy, survival

## Abstract

Upper para-aortic lymph node dissection (PALND) is one of the most challenging gynecologic robotic procedures. This study aimed to evaluate the oncologic and operative outcomes of robotic staging surgery, including upper PALND, using low pelvic port placement (LP3) in 22 patients with high-risk endometrial cancer. High-risk was defined as patients who showed deep myometrial invasion with grade III, cervical involvement, or high-risk histology. The mean patient age and body mass index were 58 years and 24 kg/m^2^. The mean operative time was 263 min. The mean number of total LNs and upper PALNs obtained was 31 and 10. Two patients received lymphangiography to reduce the amount of drained lymphatic fluid after surgery. The recurrence rate was 13.6% (3/22). There were two LN recurrences and one at the peritoneum in the intra-abdominal cavity. Robotic staging surgery using LP3 was feasible for performing PALND as well as procedures in the pelvic cavity simultaneously. It provides important techniques for performing optimal surgical procedures when surgeons decide to perform comprehensive PALND in instances of isolated recurrence or unexpected LN enlargement as well as high-risk endometrial cancer. Consequently, surgeons can achieve surgical consistency and reproducibility for PALND, leading to improved operative and survival outcomes in high-risk endometrial cancer.

## 1. Introduction

Surgery has an essential role in the management of endometrial cancer, which is the most common malignant disease of the female reproductive organ in developed countries [1]. The surgical treatment for endometrial cancer consists of a hysterectomy, often along with a salpingo-oophorectomy, and the removal of lymph nodes (LNs). Although the extent of surgery depends on the preoperative tumor stage, risk factors, or surgeons’ policies, minimally invasive surgery is regarded as a gold standard for surgery in endometrial cancer. Robotic surgery has been performed widely in gynecologic cancers as well as benign disease as a minimally invasive approach. Recently, sentinel lymph node (SLN) mapping or hysterectomy without LN dissection (LND) has also been performed in low-risk endometrial cancer [2]. Regardless of the controversies over whether SLN mapping is feasible in high-risk endometrial cancer, upper para-aortic LND (PALND) to the infrarenal level can be utilized for staging in high-risk endometrial cancer. In addition, surgeons may decide to perform comprehensive PALND in instances of isolated recurrence or unexpected LN enlargement. In spite of the advanced technical advantages of robotic surgery, upper PALND is one of the most challenging robotic procedures due to the limited range of motion of robotic arms.

We have already introduced robotic low pelvic port placement (LP3) which can be performed optimally and simultaneously with both upper PALND and pelvic procedures in endometrial cancer using the da Vinci Xi system [3]. For Asian women, who have comparably narrow body habitus, we modified the LP3. We tended to perform pelvic procedures easily after PALND was completed by higher ports compared to the conventional LP3. The aim of this study was to evaluate the oncologic and operative outcomes of robotic staging surgery using LP3 in patients with high-risk endometrial cancer.

## 2. Materials and Methods

### 2.1. Patient Cohort

From August 2019 to July 2024, 102 endometrial cancer patients underwent staging surgery by a single surgeon (J. Paek). Of these, 82 and 20 patients received robotic and open surgery, respectively. During this period, all consecutive 22 high-risk endometrial cancer patients who underwent comprehensive robotic staging surgery, including upper PALND, were analyzed. Some patients, who underwent open staging surgery due to peritoneal carcinomatosis or a bulky uterine mass more than 10 cm, were not included in this study. We performed preoperative pelvic magnetic resonance imaging (MRI) to determine the presence of high-risk factors. Preoperative high-risk was defined as endometrial cancer patients with deep myometrial invasion with tumor grade III, cervical involvement, or non-endometrioid histology. The patient’s status was estimated prospectively in terms of characteristics, operative outcomes, pathologic results, and survival outcomes. Patient characteristics included age, body mass index, ASA (The American Society of Anesthesiologists) classification, and a history of previous abdominal surgery, including both laparoscopic and abdominal approaches. Operative outcomes included operating time, perioperative blood loss, days of hospitalization, and perioperative complications. The operating time was defined as the time from the first incision to the closure of the incision. For surgical complications, we used the Clavien–Dindo classification [4]. Briefly, grade I included deviation from the normal postoperative course without pharmacological treatment or surgical interventions. Grade II included cases requiring blood transfusion as well as pharmacological treatment. Grade III included cases requiring surgical intervention. Pathologic results included tumor stage, tumor grade, tumor size, cell type, lymphovascular space invasion, positive peritoneal cytology, lymph node metastases, and postoperative adjuvant treatment. All continuous data were expressed as mean ± standard deviation, and categorical data were reported as absolute numbers or percentages. The data were analyzed using SAS/STAT software, version 9.4 (SAS Institute Inc., Cary, NC, USA). This research was approved by the Institutional Review Board of Ajou University Hospital.

### 2.2. Surgical Procedures

**Figure 1:**
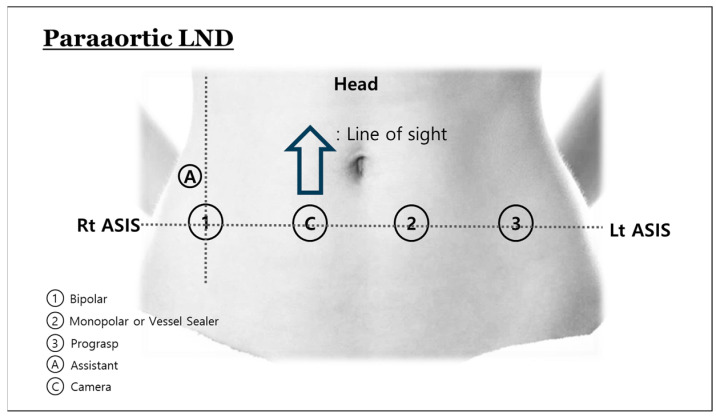
Port placement for para-aortic lymph node dissection.

**Figure 2:**
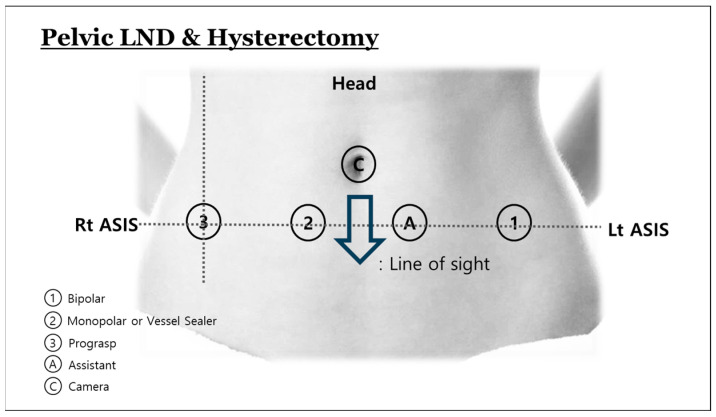
Port placement for pelvic lymph node dissection and hysterectomy.

The da Vinci Xi Surgical System (Intuitive Surgical, Inc., Sunnyvale, CA, USA) was used. The overall procedure is composed of two parts. One consists of upper abdominal procedures, including upper PALND to the infrarenal level. The other consists of pelvic procedures, including hysterectomy, bilateral salpingo-oophorectomy, and pelvic LND (PLND). No uterine manipulator is used. Considering that Asian woman have comparably narrow body habitus, we modified the location of port placement from the previous LP3 [3]. Four robotic ports are placed along a line drawn between both anterior superior iliac spines (Figure 1). An additional laparoscopic port is used for an assistant. The Supplementary Video File showed the surgical procedures. Initially, for the PALND, we reposition the small intestines to the right by rotating them counterclockwise, using Prograsp and fenestrated bipolar forceps. An incision is created in the retroperitoneum above the aortic bifurcation, located between the small intestine mesentery and sigmoid mesentery, and then extended to the duodenal folds. This allows us to distinctly visualize the left renal vein. After confirming the left renal vein, a fan retractor holds the small bowel upwards to ensure a safe operative field. The lymphatic channels are dissected bluntly, during which the right ureter is identified. With the use of a vessel sealer and bipolar forceps, we conduct the right PALND up to the renal vein level, along with aortocaval lymph node dissection. The left PALND proceeds with the use of a comparable method. After PALND is completed, the boom of robotic system is rotated 180 degrees to retarget for the pelvic procedures including the hysterectomy and PLND. Using a technique called ‘port hopping’, the robotic ports remain in the same locations, but the instruments, including the camera, are docked in different configurations (Figure 2). The hysterectomy and PLND are performed in the usual manner. The colpotomy is performed intracorporeally in all patients.

## 3. Results

A summary of subject clinical characteristics and operative outcomes is described in Table 1. The operation was completed robotically without any complications and conversions to laparotomy. The mean patient age and body mass index were 57.8 years and 24 kg/m^2^, respectively. The mean operating time was 262.5 min. The median postoperative hospital stay was 6.6 days. The mean number of total LNs and upper PALNs obtained was 31 and 10, respectively. There were no patients who needed blood transfusion. Two patients received lymphangiography to reduce the amount of drained lymphatic fluid after surgery.

A summary of subject clinicopathologic characteristics is described in Table 2. About 82% (18/22) of the analyzed patients showed deep myometrial invasion and tumor grade III in pelvic MRI and endometrial biopsy before staging surgery. Of these, seven patients had less than half of myometrial invasion on the final pathologic results. Five patients who had mesonephric carcinoma or carcinosarcoma received systemic chemotherapy with paclitaxel and carboplatin. The recurrence rate was 13.6% (3/22). There were two LN recurrences and one at the peritoneum in the intra-abdominal cavity. Table 3 shows a summary of all three patients who experienced recurrence.

## 4. Discussion

LND remains a cornerstone of surgical management for endometrial cancer, playing a crucial role in staging, prognosis, and adjuvant therapy decisions. However, advances in surgical techniques and evolving understanding of the disease have led to ongoing discussions and controversies surrounding the role of LND in endometrial cancer treatment. Recent studies have questioned the traditional belief in the survival benefits of systematic LND. The ASTEC trial and the subsequent SEPAL study provided conflicting evidence. The former showed no overall survival benefit, whereas the latter suggested a potential benefit in high-risk patients with PLND and PALND [5,6]. Despite these mixed results, the importance of adequate staging through LND remains, as it may provide crucial prognostic information, guide adjuvant therapy decisions, and improve tailored patient management. PALN metastasis has been increasingly recognized as a common site of metastasis in high-risk endometrial cancer. Several studies showed that the incidence of PALN metastasis can significantly affect prognosis and management strategies [7,8]. PALND, in conjunction with PLND, provides more comprehensive staging, which is particularly critical for high-risk patients, as it may uncover metastasis that would otherwise remain undetected [8,9]. This more thorough staging helps accurately stratify patients for appropriate adjuvant therapy and potentially improves survival outcomes.

Another key controversy surrounding LND in endometrial cancer is the extent of LND required. While extensive LND historically aimed to maximize the detection of involved LNs, recent evidence suggests that a more limited approach may be sufficient in certain low-risk cases [10]. This raises questions about the balance between accurate staging and potential overtreatment. The invasiveness of full LND has significant implications for patient morbidity, including lymphedema, vascular injury, and prolonged recovery times [11]. The increased awareness of these risks has intensified the evaluation of the necessity of such procedures in all patient subsets. Recent guidelines suggest a more selective approach, reserving extensive LND for cases with clinically suspicious nodes or high-risk histologic features. The introduction of SLN mapping has emerged as a less invasive alternative with promising results [12,13]. Studies have shown that SLN mapping accurately identifies nodal metastasis with reduced morbidity compared to systematic dissection. The FIRES trial, for example, demonstrated that SLN mapping had a high sensitivity for detecting metastases [14]. This technique is rapidly gaining acceptance, particularly for low- to intermediate-risk patients, endorsing a shift towards more personalized surgical approaches.

The utilization of robot-assisted surgery for lymphadenectomy in endometrial cancer has demonstrated several advantages over traditional laparoscopic or open techniques [15]. Recent studies have shown that robotic lymphadenectomy is associated with reduced blood loss, shorter hospital stays, and faster recovery times compared to open surgery. Moreover, robotic surgery allows for greater precision and dexterity within the narrow confines of the pelvic cavity, potentially leading to a more thorough dissection and improved lymph node yield [16,17]. Studies have indicated that robotic lymphadenectomy results in similar oncologic outcomes compared to conventional methods, with no significant differences in recurrence rates or overall survival [15,16,17,18]. This makes robotic surgery an attractive option for endometrial cancer patients in need of LND, particularly those who may benefit from minimally invasive procedures due to comorbid conditions or obesity.

Robotic PALND has emerged as a feasible and effective technique in the management of endometrial cancer, particularly when extensive LND is required [19]. The complexity of PALND, especially in the upper para-aortic region, poses significant challenges due to the intricate anatomy and confined space. Recent reports have shown that robotic systems can enhance the surgeon’s ability to perform meticulous dissections in these challenging areas [20,21]. Although robotic approach enables better visualization and access and minimizes the need for extensive bowel mobilization, robotic upper PALND is one of the most challenging gynecologic procedures. The robotic staging surgery using LP3 makes it easy to reach up to the renal vessels and perform comprehensive lymphadenectomy without complications. The LP3 is enhanced by the advanced technology of the Xi system, which includes a patient clearance function, a rotating boom, and ‘port hopping’, which enables any port to be used for the camera [3]. Furthermore, compared to conventional port placement, the LP3 allows for safer angles with large vessels and provides sufficient distance to access targets in the upper abdomen. Some clinical studies indicate that there is no set number of LNs required for effective sampling, but the sensitivity of LND increases with the removal of more nodes. Chan et al. suggest that a resection of 21 to 25 nodes results in an 80% probability of detecting at least one positive LN [21]. Meanwhile, Mayo Clinic’s guidelines recommend retrieving at least 22 pelvic and 10 para-aortic nodes for diagnostic adequacy [22]. Additionally, studies by Cragun et al. and Lutman et al. highlight that retrieving a higher number of nodes correlates with improved survival outcomes in high-risk endometrial cancer patients [23,24]. Given this context, our robotic staging surgery using the LP3 in high-risk endometrial cancer demonstrates that our LN retrieval is sufficient, highlighting the stability and effectiveness of robotic lymphadenectomy.

These advancements improve surgical outcomes, especially for high-risk patients who may have extensive nodal disease, leading to greater surgical consistency and reproducibility and, ultimately, improved survival outcomes. While the debate on the role of LND in endometrial cancer continues, a one-size-fits-all approach is no longer tenable. Personalized medicine, supported by techniques such as SLN mapping and comprehensive molecular profiling, is increasingly guiding the future landscape of surgical oncology [22]. Ongoing research and clinical trials will be pivotal in refining the role of LND, ensuring a balanced approach that maximizes therapeutic benefits while minimizing surgical risks.

## 5. Conclusions

Robotic staging surgery using LP3 in patients with high-risk endometrial cancer was feasible for performing PALND as well as procedures in the pelvic cavity simultaneously. It provides important techniques for performing optimal surgical procedures when surgeons decide to perform comprehensive PALND in instances of isolated recurrence or unexpected LN enlargement as well as high-risk endometrial cancer. Consequently, surgeons can achieve surgical consistency and reproducibility for PALND, leading to improved operative and survival outcomes in high-risk endometrial cancer.

## Figures and Tables

**Table 1:** Patient characteristics and operative outcomes.

	Patients (*n* = 22)
Age (years ± standard deviation [SD])	57.8 ± 7.1
Body mass index (kg/m^2^ ± SD)	24.0 ± 3.9
ASA classification	
I (normal healthy patient)	8 (36.4%)
II (mild systemic disease)	14 (63.6%)
Previous abdominal surgery history	11 (50%)
Peritoneal adhesion	9 (40.9%)
Operating time (min ± SD)	262.5 ± 18.9
Estimated blood loss (mL ± SD)	72.7 ± 34.5
Postoperative hemoglobin drop (g/dL ± SD)	1.4 ± 0.7
Days of hospitalization (±SD)	6.6 ± 3.2
Number of lymph nodes retrieved (±SD)	30.9 ± 13.7
Number of para-aortic lymph nodes retrieved (±SD)	10.2 ± 5.1
Perioperative complications (%)	
Grade I	1 (4.5%)
Grade II	0
Grade III	2 (9.1%)

**Table 2:** Clinicopathologic characteristics.

	Patients (*n* = 22)
Preoperative high-risk factor	
Deep myometrial invasion with tumor grade III	18 (81.8%)
Cervical involvement	2 (9.1%)
Non-endometrioid histology	5 (22.7%)
Tumor stage	
IA	7 (31.8%)
IB	7 (31.8%)
II	2 (9.1%)
IIIA	3 (13.6%)
IIIB	0
IIIC1	1 (4.5%)
IIIC2	2 (9.1%)
Tumor grade	
I	3 (13.6%)
II	1 (4.5%)
III	18 (81.8%)
Histology	
Endometrioid	17 (77.3%)
Mesonephric	3 (13.6%)
Carcinosarcoma	2 (9.1%)
Tumor size (cm ± SD)	2.6 ± 1.3
Lymphovascular space invasion	7 (31.8%)
Positive peritoneal cytology	5 (22.7%)
Pelvic lymph node metastases	3 (13.6%)
Para-aortic lymph node metastases	2 (9.1%)
Postoperative adjuvant treatment	17 (77.3%)
Vaginal brachytherapy	4 (18.2%)
Extended beam radiotherapy (EBRT)	8 (36.4%)
Chemotherapy	3 (13.6%)
Chemotherapy + EBRT	2 (9.1%)
Recurrence	3 (13.6%)

**Table 3:** Clinicopathologic characteristics of patients with recurrence.

**Patients**	**#1**	**#2**	**#3**
Site of recurrence	Para-aortic LN	Peritoneum	Cardiophrenic LN
Recurrence (months)	12	29	12
Death (months)	−	−	−
Treatment after recurrence	Chemotherapy after surgery	Chemotherapy	Chemotherapy after surgery
Age (years)	52	54	50
Tumor stage	IIIA	IA	IIIC1
Tumor grade	II	III	III
Histology	Endometrioid	Carcinosarcoma	Mesonephric
Tumor size (cm)	1	3	7
Lymphovascular space invasion	+	−	+
Positive peritoneal cytology	+	−	+
Lymph node metastases	−	−	+ (Pelvic)
Primary adjuvant treatment	EBRT	EBRT	Chemotherapy

## Data Availability

Data are contained within the article.

## Supplementary Materials

The following supporting information can be downloaded at: https://www.mdpi.com/article/10.3390/curroncol31120576/s1.
